# Comparative Property Analysis of One-by-One Rib Lingerie Fabrics Fabricated from Modal Fibers and Microfibers

**DOI:** 10.3390/nano15090653

**Published:** 2025-04-25

**Authors:** Antoneta Tomljenović, Juro Živičnjak

**Affiliations:** Department of Materials, Fibers and Textile Testing, University of Zagreb Faculty of Textile Technology, Prilaz baruna Filipovića 28a, 10000 Zagreb, Croatia; juro.zivicnjak@ttf.unizg.hr

**Keywords:** microfiber, modal, modal-micro, yarn type, rib knitted fabric, lingerie, physical and wearing properties

## Abstract

Although the applicability of modal fibers and microfibers for the production of lingerie knitwear is confirmed by commercial use, their share in the total consumption of man-made cellulosic fibers is very low. Their applicability in the fabrication of one-by-one rib weft-knitted fabrics, as well as comparative analyses of the influence of differently spun modal and modal-micro yarns on physical, usage, esthetic and wearing comfort properties have not been sufficiently investigated. In this study, a comparative analysis of innovative rib knitted fabrics made of regular–fine modal fibers (1.3 dtex) and 1.0 dtex microfibers is therefore carried out to determine their properties at different relaxation stages. For this purpose, two lines of one-by-one rib fabrics were fabricated from ring-, air-jet- and open-end rotor-spun modal and modal-micro yarns in the same way. The results showed that rib lingerie fabrics fabricated from modal microfibers are lighter and thinner, have a higher voluminosity and moisture absorption capacity, and consequently have slightly lower porosity, breathability and abrasion resistance than fabrics made from modal regular–fine fibers, as well as comparable dimensional stability, tensile strength and pilling properties, but mainly after a wet relaxation treatment.

## 1. Introduction

Textile microfibers are man-made fibers that can be produced to be finer than any natural fiber, in the range of 0.1 to 1.0 dtex. They are usually synthetic fibers made of polyamide, acrylic and polyester, or artificial fibers made of cellulose such as modal, viscose and lyocell, which are produced in the fineness range of 0.5 to 1.0 dtex [1]. Microfibers, alone or in blends, are used in many clothing lines, as they offer better properties in terms of wearing quality and comfort, a luxurious look due to a smaller diameter, a larger surface area and flexibility compared to conventional textile fibers [1,2,3].

Artificial fibers made from pure cellulose are preferred as a substitute for cotton, especially for lingerie knitwear worn directly on the skin [4]. One of these, modal fibers, are fabricated using a modal process (modified viscose process) that significantly increases the tensile strength and wet modulus compared to conventional viscose. In addition, modal fibers have a higher degree of cellulose polymerization, higher crystallinity, a more pronounced fibrillary structure, a more regular cross-sectional shape and lower swelling, but ensure high moisture absorption capacity, efficient moisture management, improved breathability, natural softness and exceptional contact comfort [5,6].

In an analysis of global fiber production in 2023, cellulosic man-made fibers, including viscose, acetate, lyocell, cupro and modal, had a market share of around 6% with an annual production volume of around 7.9 million tons. Viscose accounts for the majority of the global market for cellulosic man-made fibers (80%), acetate has a market share of around 13%, lyocell around 4%, cupro around 0.2% and modal fibers (including microfibers), despite their improved properties, account for around 3%, with a production volume of only 0.2 million tons [7,8,9].

The finer the modal microfibers are, the weaker they become, but the strength of the yarn generally increases because many microfibers are packed very densely in the yarn with the same linear density. The irregularity of this yarn generally improves and the number of thin places and hairiness decrease, while the number of neps and thick places increase [1,10,11,12].

However, ring spinning, open-end rotor spinning and air-jet spinning are three important production systems for yarns made from modal staple fibers and microfibers, each of which produces yarns with different structures and physical–mechanical properties [1]. In conventional ring-spun (RiS) yarns, the fibers are arranged in a helix and the resulting yarn has a uniform fiber core. In unconventional open-end rotor-spun (RoS) yarns, the core fibers that form the inner part of the yarn are twisted and some surface fibers are wrapped around the core. Air-jet-spun (AiS) yarns have a core where the fibers run parallel to the yarn axis, without any twist, resulting in wrapping of the core by the surface fibers along the length [13,14,15].

The structural differences between ring-, open-end rotor- and air-jet-spun yarns produced from modal fibers and microfibers therefore also affect their quality and have already been explained [16]. When comparing yarns with the same linear density, the highest strength was achieved with ring-spun yarns, followed by air-jet- and open-end rotor-spun yarns consisting of both modal fibers and microfibers. The values of overall unevenness of the differently spun modal yarns are greater than or equal to the same values of the modal microfibers. The hairiness in yarns increases as follows: AiS yarns < RoS yarns < RiS yarns, with finer fibers causing lower hairiness in all yarn types.

Weft-knitted fabrics, usually in single- and double-jersey patterns, are most commonly used for lingerie, as they have better elasticity compared to woven fabrics, which is very important for freedom of movement [17]. There are numerous studies comparing different properties of weft-knitted fabrics fabricated of cotton and various cellulosic man-made fibers (including modal and/or modal microfibers). They mainly refer to knitted fabrics finished with silicone softeners [10,11] and treated with formic acid [18], or to the evaluation of the influence of the fiber type on the structure of the knitted fabric, tensile or burst strength, moisture and water absorption, air permeability, thermal properties, ultraviolet protection, abrasion resistance and bending stiffness [12,19,20]. It is particularly important to point out that all the above analyses were carried out on plain single-jersey weft-knitted fabrics. When analyzing the thermal and moisture comfort of knitwear for lingerie, those made of modal are generally rated as better [21].

In general, microfiber knitwear has proven to be physiologically advantageous to wear as it is more voluminous, softer, smoother and has better moisture regulation; above all, it is lightweight and has excellent fit and shape retention [1]. A review of the literature revealed a smaller number of comparative studies on the specific structural, physical, mechanical and wearing properties of knitwear fabricated from cellulosic microfibers. For example, single-jersey weft-knitted fabrics made from viscose microfibers were found to have a higher stitch density and tightness factor, better burst strength, dimensional stability, drapability and less spirality than the same fabrics made from regular–fine viscose fibers [22]. In a comparison of plain single-jersey weft knits made of modal fibers and microfibers, it was found that fabrics with microfibers have a lower thickness, shrinkage and air permeability, as well as a higher burst strength, pilling tendencies and better thermal comfort [2]. The structural, physical, mechanical and thermal properties of single-jersey weft-knitted fabrics fabricated from a 50/50 blend of modal microfibers with cotton and a 50/50 blend with regular–fine modal fibers were also evaluated [3].

Research on weft knits fabricated of modal fibers in other patterns such as single-pique and honeycomb [23,24,25], and especially one-by-one rib (or rib jersey), is less common [26], although the stretchability and elasticity of one-by-one rib knitted fabrics under the same conditions is higher than that of single jersey [27], which is of particular importance for lingerie items (e.g., T-shirts, undershirts, underpants and nightwear).

The properties of the differently spun yarns have a considerable influence on the properties of weft-knitted fabrics [28]. The literature search also revealed that there is a lack of comparative analyses on the influence of differently spun modal and modal-micro yarns on the wearing properties of lingerie knitted fabrics, even when produced in a single-jersey pattern [13,29,30].

Many properties determine the wearing quality of weft knits and thus also the quality of the underwear for which it is used [4,23]. Knitted fabrics made from man-made cellulosic fibers and microfibers are usually tested at different stages of relaxation to ensure the quality of fabrication: after knitting, when they have been laid flat freely for sufficient time and have reached the dry relaxed state, and optionally in the fully relaxed state after a wet treatment and drying [2,3,4,28,31], where the forces required to keep the yarn in loop form are reduced. Accordingly, the structural parameters of knitted fabrics change and thus so do their physical properties, which have a direct effect on the wearing properties of knitwear [4]. These include, in particular, bursting strength, tensile strength and elongation, abrasion resistance, dimensional stability in domestic laundering and pilling tendency, which are responsible for usability and esthetics, as well as breathability and moisture absorption, related to the wearing comfort of lingerie.

It turns out that despite the progress made in researching modal fibers and microfibers for the production of lingerie knitted fabrics worn directly on the skin, and despite their applicability confirmed by commercial use, their share in the total consumption of cellulosic man-made fibers is very low. A review of the literature revealed that their applicability in the fabrication of one-by-one rib weft-knitted fabrics and comparative analyses of the influence of differently spun modal and modal-micro yarns on the wearing properties of rib lingerie items have not been sufficiently researched. The aim of this work is therefore to carry out a comparative analysis of innovative rib knitwear with advanced properties made from regular–fine modal fibers (1.3 dtex) and 1.0 dtex fine microfibers, based on previously published studies [4,30], in order to determine and compare their physical and wearing properties in different relaxation stages to ensure the quality of fabrication. Accordingly, two lines of one-by-one rib fabrics were fabricated from ring-, air-jet- and open-end rotor-spun modal and modal-micro yarns under the same conditions. The influence of the structure and physical properties of the differently spun yarns and the fabricated lingerie fabrics on their wearing properties was analyzed by determining their tensile strength and elongation, abrasion resistance, dimensional stability in household laundry, pilling tendency, breathability and moisture absorption capacity.

## 2. Materials and Methods

### 2.1. One-by-One Rib Knit Fabric Fabrication

Two lines of circular lingerie weft-knitted fabric were fabricated in a one-by-one rib pattern that consisted of 1 × 1 rib courses, producing single wales of face stitches alternating with single wales of reverse stitches (EN ISO 8388:2003) [32], using a double-bed circular knitting machine with gauge E17, eight knitting systems and a needle bed diameter of 200 mm (including 432 × 2 needles). Three types of single-spun modal (Md) and modal-micro (Mdm) yarns—ring-spun (RiS), open-end rotor-spun (RoS) and air-jet-spun (AiS)—were used for knitting. Therefore, six different rib fabrics were knitted from a single modal or modal-micro yarn under the same conditions. All yarns with a nominal linear density of 20 tex were spun in the spinning mill in Klanjec, Croatia, from LenzingTM Modal and LenzingTM Modal Micro bright staple fibers, both with a length of 38/40 mm and a regular fineness of 1.3 dtex for modal fibers and 1.0 dtex for modal microfibers, whereby the optimum yarn linear density range that could be achieved with each spinning method varied within 20 ± 0.4 tex. A detailed analysis of the physical–mechanical properties of the modal and modal-micro yarns used has already been published [16], and the selected ones are shown in Table 1.

### 2.2. One-by-One Rib Knit Fabric Relaxation Treatments

After the lingerie knitted fabrics were removed from the knitting machine, to ensure the quality of their fabrication and to equalize their dimensions, they were subjected to two relaxation processes: dry relaxation and wet relaxation to achieve the complete state of relaxation. To achieve the dry relaxation state, the knitted fabrics were left to lie freely on the flat surface of the table for approximately 96 h in a standard atmosphere with a temperature of 20 ± 2 °C and a relative humidity of 65 ± 4% in accordance with EN ISO 139:2005/A1:2011 [33]. For complete relaxation, the knitted fabrics were first washed in a bath at an initial temperature of 40 °C with the addition of 1 g L^−1^ wetting agent for 15 min, and then with the addition of hydrogen peroxide (2 g L^−1^) and stabilizer (2 g L^−1^) at 95 °C for 45 min at a pH value of 10.5. The knitted fabrics were then rinsed at 80 °C for 10 min, rinsed cold for 10 min with the addition of acetic acid to a neutral pH value and softened with silicone softener (2% of the fabric weight). They were then dried through the dryer at a temperature of 150 °C with a throughput speed of 0.15 m s^−1^ and conditioned for 24 h under standard atmospheric conditions [33].

### 2.3. One-by-One Rib Knit Fabric Property Analysis Methodology

**Figure 1:**
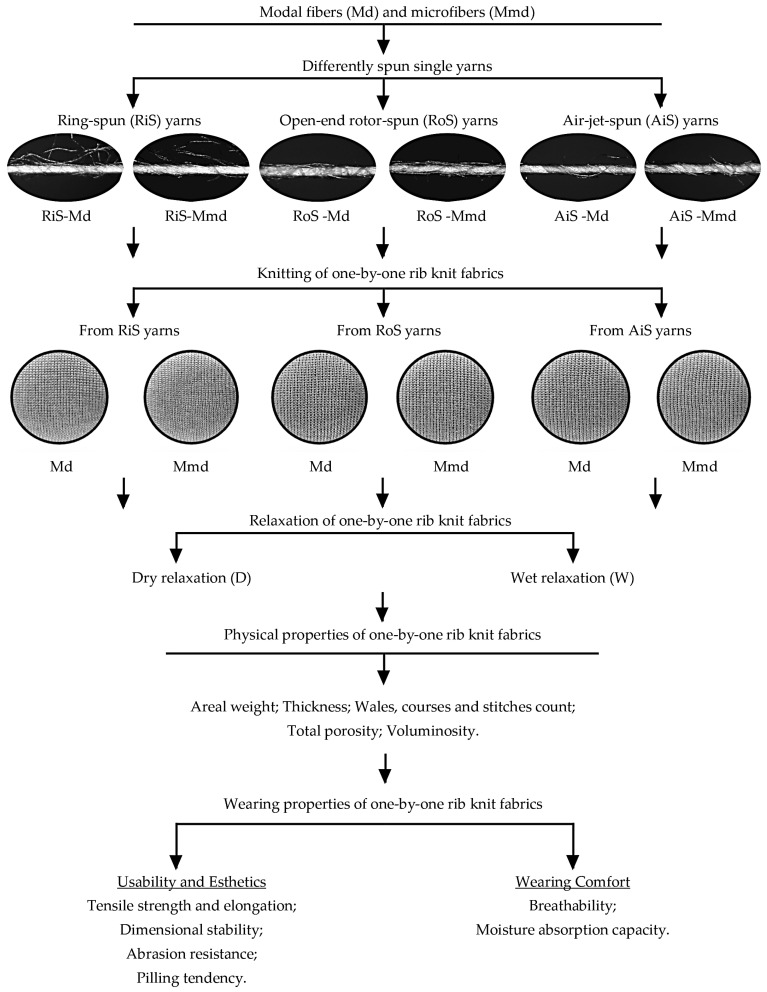
Experimental design carried out.

In order to analyze the differences between one-by-one rib knitted lingerie fabrics fabricated from differently spun single yarns of regular–fine modal fibers and microfibers, the influence of fiber, yarn and knit relaxation type on their properties was evaluated according to the experimental design shown in Figure 1.

#### 2.3.1. Physical Properties of Knitted Fabrics

The one-by-one rib lingerie fabrics were comparatively analyzed on the basis of the following measured and calculated physical properties: areal weight, thickness, wale, course and stitch counts, total porosity and voluminosity.

The areal weight of the ribbed knitted fabric was calculated in g m^−2^ using five measurements on round specimens in g cm^−2^ and multiplied by 100 according to EN 12127:2003 [34]. The thickness of the ribbed knitted fabric in mm was measured ten times under a pressure of 1 kPa, with the thickness gage 2000-U (Hess MBV GmbH, Sonsbeck, Germany) according to EN ISO 5084:2003 [35]. The number of wales and courses per cm of the ribbed knitted fabric and the stitches per cm^2^ were measured five times according to EN 14971:2008 [36]. The total porosity of ribbed knitted fabrics in %, defined as the ratio of all air spaces in the fabric both between yarns and inside them [37], was calculated using Equation (1):

Rib knit total porosity (%) = (Fiber density (g cm^−3^) − Rib knit voluminosity (g cm^−3^)/Fiber density) × 100 (1)

where a value of 1.5141 g cm^−3^ [38] was used as the modal fiber density, and the voluminosity of the ribbed knitted fabrics in g cm^−3^ was calculated using Equation (2):

Rib knit voluminosity (g cm^−3^) = Rib knit areal weight (g m^−2^)/1000 × Rib knit thickness (mm) (2)

#### 2.3.2. Wearing Properties of Knitted Fabrics

The following wearing properties, which are responsible for usability and esthetics, were analyzed comparatively on one-by-one rib lingerie fabrics: tensile strength and elongation, dimensional stability in domestic laundering, abrasion resistance and pilling tendency, and breathability and moisture absorption capacity, which are responsible for wearing comfort.

Since the knitted fabrics for lingerie items generally exhibit up to four times greater elongation at break in the course direction [26], the breaking strength in N and the elongation at break in % of the ribbed knitted fabric were measured separately for the wale and course directions, using the Tensolab 3000 strength tester, Mesdan S.p.A., Raffa, Italy, according to EN ISO 13934-1:2013 [39]. Five strip specimens measuring 200 mm × 50 mm were tested at a tensile speed of 100 mm/min until breakage in both directions.

The dimensional stability of circular ribbed knitted fabrics in the wale and course directions was determined according to EN ISO 6330:2012 [40] after a mild household wash cycle in an Elecrolux Wascator FOM1 CLS at 40 °C using a phosphate-free ECE reference detergent without optical brighteners (SDC Enterprises Limited, Holmfirth, UK), Sweden, and after air drying. The dimensional changes in % for both directions were evaluated according to EN ISO 3759:2011 and EN ISO 5077:2008 [41,42] using Equation (3):

Dimensional change (%) = (Length after laundering (mm) − Initial length (mm)/Initial length (mm)) × 100(3)

The abrasion resistance and pilling tendency of the ribbed knitted fabric were determined using the Martindale abrasion and pilling tester, Mesdan S.p.A., Italy, following EN ISO 12947-2:2016 [43] and EN ISO 12945-2:2020 [44]. The abrasion properties and the tendency to pill on the surface of the knitted fabric were also evaluated on three separately tested specimens rubbed against a reference wool abrasive (SDC Enterprises Limited, UK). Abrasion resistance was expressed as the number of abrasion rubs at which breakage occurred (with evaluation intervals from 6001 to 20,000 abrasion rubs every 2000 abrasion rubs and from 20,001 to 40,000 abrasion rubs every 5000 abrasion rubs), while pilling tendency was assessed visually by three observers according to EN ISO 12945-4:2020 [45]. After a certain number of pilling rubs (125, 500, 1000, 2000, 5000 and 7000), ratings from 5 to 1 (5—no change, to 1—strong change) were assigned using the Roaches SM54 knitted standards, double jersey, UK.

The breathability of the ribbed knitted fabric was measured according to EN ISO 9237:1995 [46] using the air permeability tester (Air Tronic Mesdan S.p.A., Puegnago del Garda, Italy). The airflow through the tested knitted fabric was measured ten times under a pressure of 100 Pa and the breathability was calculated using Equation (4):

Rib knit breathability (dm^3^ min^−1^ cm^−2^) = Air flow through the knit (dm^3^ min^−1^)/Knit tested area (5 cm^2^) (4)

The moisture absorption capacity in % of the ribbed knitted fabric was determined three times per knitted sample following ASTM D 2654-89a [47]. The conditioned weight [33] and the weight for the absolutely dry state of three specimens per knitted sample were measured, and the moisture absorbency was calculated according to Equation (5):

Knit absorption capacity (%) = (Weight _conditioned_ (g) − Weight _absolutely dried_ (g)/Weight _absolutely dried_ (g)) × 100 (5)

where applicable, the average result and the standard deviation were calculated for all properties determined.

## 3. Results and Discussion

The results contain a comprehensive comparative analysis of the physical and wearing properties of two innovative one-by-one rib lingerie fabric lines fabricated from differently spun yarns made of regular–fine modal fibers (1.3 dtex) and 1.0 dtex fine microfibers after two different relaxation processes. The advances in the application of modal fibers and microfibers were discussed by analyzing the influence of fiber fineness, the different types of single-spun yarns and the relaxation phase of ribbed knitted fabrics on their properties to confirm their quality.

### 3.1. Comparative Analysis of One-by-One Rib Knit Physical Properties

The results for the areal weight, thickness, and wale and course counts of the dry and wet relaxed ribbed knitted fabrics are shown in Table 2, while the results for stitch count, voluminosity and total porosity are shown in Table 3.

Although all ribbed lingerie fabrics are knitted with the same parameters on the same machine, the results of their physical properties differ from one another. This is reflected above all in the measured values of areal weight, thickness and wale and course counts of the dry relaxed rib knit samples (Table 2).

From the results shown in Table 2, it can be seen that the areal weight values and subsequently the thickness of the dry relaxed ribbed knitted fabrics fabricated from modal fibers and microfibers depend directly on the structure of the knitted fabric, primarily on the number of wales and courses per cm, which is then reflected in the calculated values of stitches per cm^2^ (Table 3), as well as on the unevenness of the yarns used for their production. Simultaneously, the areal weight of rib knitted fabrics increases as follows: RoS knitted fabrics < AiS knitted fabrics < RiS knitted fabrics, both in the modal and modal-micro samples of dry relaxed rib knitted fabrics. The dependence of the overall yarn unevenness values of differently spun yarns made from modal fibers and microfibers (Table 1) is shown by the fact that knitted fabrics made from yarns with higher evenness have higher areal weight values (Table 2). At the same time, the overall unevenness values of the yarns used increase as follows: RiS yarns < AiS yarns < RoS yarns (Table 1).

This traceability can also be seen in the results of the thickness of the dry relaxed knitted samples (Table 2).

It follows that lower values for overall yarn unevenness affect the higher number of knit wales per cm (Table 2), which is highest for dry relaxed knits fabricated from ring-spun yarns in both modal and modal-micro ribbed knitted fabrics, followed by knits fabricated from air-jet-spun and open-end rotor-spun yarns. It can be seen that the number of wales per cm in all samples of dry relaxed rib knitted fabrics is higher in knitted fabrics made of modal microfibers than in knitted fabrics made of regular–fine modal fibers (Table 2). The number of courses per cm is the same for all dry relaxed modal-micro knitted fabrics (13 cm^−1^) and differs only minimally for modal knitted fabrics (12, 12.5 and 13 cm^−1^). After wet relaxation treatment, the number of courses does not change, except for the RiS-Md and AiS-Mdm knitted samples (Table 2). It turns out that the dimensional changes in knitwear after wet treatment are primarily caused by structural changes in the width direction of the knitwear (i.e., a change in wale count), which is to be expected in rib knitwear.

It is generally known that when knitting, the yarns that form the fabric are under tension. When the fabric is removed from the knitting machine, it has time to relax, as can be seen from the changed dimensions. During the relaxation process, the internal tensions decrease and the knitted fabric gradually enters a balanced state, which is influenced by two forces: the resiliency force of the stitched yarn, which tries to straighten the yarn, change its configuration and bring the knitted fabric into a balanced state, and the friction force, which overcomes the movement of the yarn in a stitch and fixes the unbalanced state [23,48]. The incompletely balanced state of the knitted fabric during dry relaxation also changes after the wet relaxation treatment, during which the knitted fabric tends to exhibit residual extension or shrinkage, which is influenced by the release of the stresses exerted on the fabrics during manufacture. At the same time, the resiliency and the frictional forces of a stitched yarn depend primarily on the fiber material used and the yarn structure. It is obvious that in tightly twisted ring-spun yarns with the highest surface hairiness, the frictional force decreases, causing the knitted fabric to expand in the width direction, while knitted fabrics made from open-end rotor-spun yarns with less twisted surface fibers shrink, and consequently the number of wales increases.

Therefore, after wet relaxation, dimensional changes occur in the ribbed knitted fabrics and the areal weight of the knitted fabrics decreases for knitted fabrics made of ring-spun yarns (by −10.9% for RiS-Md-W and by −15.2% for RiS-Mdm-W) and increases for knitted fabrics made of open-end rotor-spun yarns (by 11.2% for RoS-Md-W and by 15.2% for RoS-Mdm-W). Meanwhile, the changes are smallest for knitted fabrics made of air-jet-spun yarns (by 0.1 for AiS-Md-W and by −4.5% for AiS-Mdm-W), which indicates that this is the most stable structure of the knitted fabrics. As the areal weight of the wet relaxed fabric samples depends primarily on the change in stitch density, the highest values for areal weight (Table 2) and stitch count per cm^2^ (Table 3) were found for the knitted fabrics fabricated from open-end rotor-spun yarns, which also indicates the greatest structural changes in these knitted fabrics. Wet relaxation reduces the thickness of all knitted fabrics, with the average value for rib knitted fabrics made of modal fibers being 0.71 mm (Table 2). It should be noted that the rib knitted fabrics made from modal microfibers had a lower areal weight and a lower thickness (Table 2) after wet relaxation treatment than the same tested knitted fabrics made from regular–fine fibers. Thus, it was found that one-by-one rib knitted fabrics made of modal microfibers are thinner and lighter than comparable knitted fabrics made of modal regular–fine fibers (which corresponds to the literature data for plain single-jersey weft-knitted fabrics [1,2]), but only after their structure has been stabilized by wet treatment.

The calculated voluminosity values of the knitted fabrics depend directly on the values for areal weight and thickness of the knitted fabrics (Table 2). It follows that all tested ribbed knitted fabrics are more voluminous after the wet treatment due to the full relaxation of the structure (Table 3), with knitted fabrics made of modal microfibers having a higher voluminosity than the same type of knitted fabrics made of regular–fine fibers. This can be explained by the higher number of microfibers per yarn diameter of equal linear density (Table 1). The number of modal fibers along the yarn cross-section was between 155 and 156, while the microfibers were very densely packed and their number was higher, ranging from 200 to 202 [16]. In this context, it is worth mentioning that ribbed knitted fabrics fabricated from differently spun yarns differ. Knitted fabrics made from open-end rotor-spun yarns and air-jet-spun yarns have a higher bulkiness compared to knitted fabrics made from ring-spun yarns (Table 3), depending on the order: RiS knitted fabrics < AiS knitted fabrics < RoS knitted fabrics. It follows that rib knitted fabrics made from yarns with higher surface uniformity and tenacity (Table 1) also have a lower bulkiness.

The values of the total porosity of the dry relaxed ribbed knitted samples shown in Table 3 are relatively high (approx. 87–88%), which guarantees the wearing comfort of the lingerie items. They are inversely proportional to the voluminosity, which means that the more voluminous samples have a lower total porosity. After the wet relaxation treatment, the volume fulfillment of the rib knitwear increases and its overall porosity decreases, but up to a maximum of 3.8%. At the same time, slightly lower values were found for wet relaxed knitted fabrics made from modal microfibers than for comparable knitted fabrics made from regular–fine modal fibers. It should be noted that knitted fabrics made from open-end rotor-spun yarns have lower total porosity values than knitted fabrics made from ring-spun and air-jet-spun yarns, as follows: RiS knitted fabrics > AiS knitted fabrics > RoS knitted fabrics. This could be due to the fact that with more compact ring-spun yarns, the porosity in the yarn is lower, but the space between the yarns is larger, so that the fabric has a more open, permeable structure. In open-end rotor-spun yarns, less twisted surface fibers probably reduce the porosity between the yarns.

### 3.2. Comparative Analysis of One-by-One Rib Knit Wearing Properties

#### 3.2.1. Usability and Esthetic Properties of Knitted Fabrics

The results of the breaking strength and elongation at break of the dry and wet relaxed one-by-one ribbed knitted fabrics, which were determined for the wale and course directions, are listed in Table 4.

The results presented in Table 4 show that the breaking strength values of the dry relaxed modal and modal-micro rib knitted fabrics are much higher in the wale direction than in the course direction. The highest values were found for rib knit samples fabricated from ring-spun yarns, which then decrease as follows: RiS knitted fabrics > AiS knitted fabrics > RoS knitted fabrics. They depend primarily on the tenacity of the yarns used for their fabrication, which are shown in Table 1 (from which it can be seen that they decrease as follows: RiS yarns > AiS yarns > RoS yarns), but also on the different lengthwise structures of the knitted fabrics (in particular the number of wales per cm (Table 2)). The breaking strength values in dry relaxed rib knitted fabrics are more uniform in the course direction due to the more uniform width structure of the knitted fabrics (i.e., smaller deviations in the number course count per cm; see Table 2).

After the wet relaxation treatment, the breaking strength decreases as follows for all knit samples and amounts in the wale direction: for knitted fabrics made of ring-spun yarns, by −33.1% for modal and by −38.4% for modal-micro; for knitted fabrics made of open-end rotor-spun yarns, by −14.7% for modal and by −11.0% for modal-micro; and for knitted fabrics made of air-jet-spun yarns, by −26.0% for modal and −20.4% for modal-micro. The structural changes caused by the relaxation of the fabrics after wet treatment contribute to a greater uniformity of the breaking strength values in the wale direction for all tested samples. The decrease in breaking strength in the course direction is less pronounced for fabrics fabricated from ring-spun and rotor-spun yarns (by −9.6% for modal and by −10.5% for modal-micro; by −17.7% for modal and by −15.2% for modal-micro, respectively) and more pronounced for fabrics fabricated from air-jet-spun yarns (by −21.8% for modal and by −31.4% for modal-micro). It follows that minimal differences in breaking strength were found in comparable identical knitted fabrics fabricated from modal fibers and microfibers. Only dry relaxed rib knitted fabrics fabricated from ring-spun microfiber yarn exhibited slightly higher values in the lengthwise direction (Table 4).

When analyzing the results for elongation at break shown in Table 3, higher values were determined in the course direction of the knitted fabric than in the wale direction. Since lingerie knitwear generally exhibits up to four times higher elongation at break in the course direction [4,26], the usability of the rib fabric tested is confirmed. After wet relaxation, elongation at break values generally decrease, which is not necessarily a disadvantage as rib knitwear tends to exhibit residual elongation during wear and body movement. The reduction in elongation at break in the wale direction of the knitwear samples is less than in the course direction (Table 4), as follows: for knitwear fabricated from ring-spun yarns, by −20.2% for modal and by −26.9% for modal-micro; for knitted fabrics made from open-end rotor-spun yarns, by −25.6% for modal and by −27.0% for modal-micro; and for knitted fabrics made from air-jet yarns, by −29.3% for modal and −26.4% for modal-micro. In this case, the highest values for elongation at break for knitted fabrics fabricated from rotor-spun yarns were determined for dry and wet relaxed rib knitted fabrics tested in both directions, as follows: RoS knitted fabrics > AiS knitted fabrics > RiS knitted fabrics. This confirms that knitted fabrics with higher breaking strength have lower elongation at break. When comparing the same knitted fabrics made of modal fibers and microfibers, minimal differences in elongation at break were found for both dry and wet relaxed fabrics.

**Figure 2:**
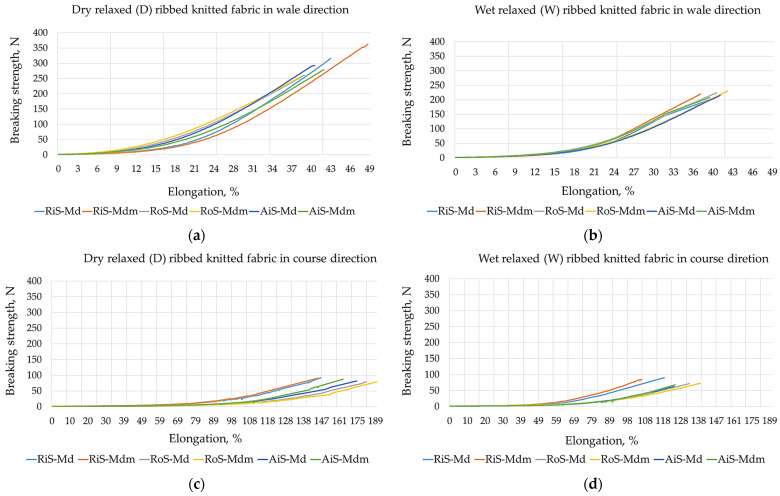
Strength/elongation diagrams for modal (Md) and modal-micro (Mdm) ribbed knitted fabrics fabricated from RiS (ring-spun), RoS (open-end rotor-spun) and AiS (air-jet-spun) yarns: (**a**) dry relaxed (D) in wale direction; (**b**) wet relaxed (W) in wale direction; (**c**) dry relaxed (D) in course direction; (**d**) wet relaxed (W) in course direction.

As a contribution to the comparative analysis of tensile strength properties, the tensile strength/elongation diagrams for all knitted fabric samples are shown in Figure 2.

**Figure 3:**
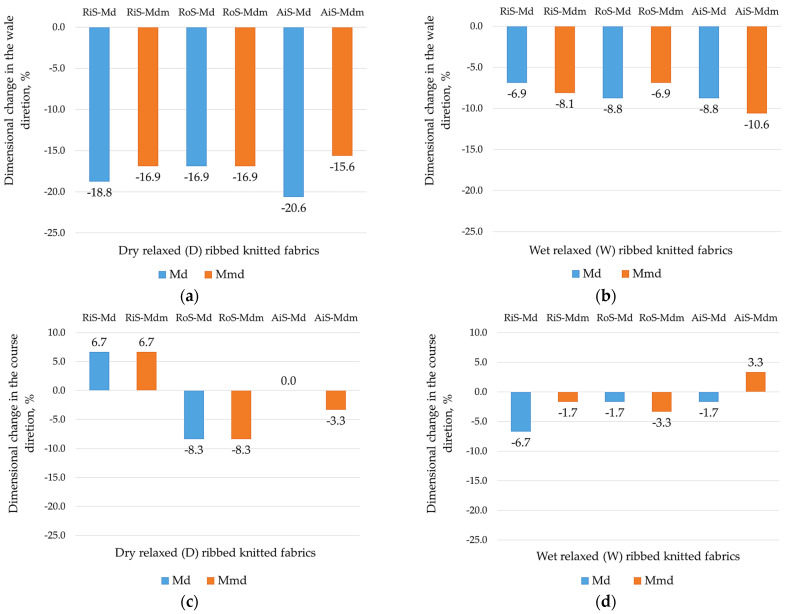
Dimensional changes in modal (Md) and modal-micro (Mdm) ribbed knitted fabrics fabricated from RiS (ring-spun), RoS (open-end rotor-spun) and AiS (air-jet-spun) yarns: (**a**) dry relaxed (D) in wale direction; (**b**) wet relaxed (W) in wale direction; (**c**) dry relaxed (D) in course direction; (**d**) wet relaxed (W) in course direction.

After domestic laundering and drying, knitted fabrics in rib patterns are particularly susceptible to dimensional changes [4], which has a direct impact on their usage and esthetic properties. Figure 3 therefore shows the results of the dimensional changes in the wale-wise direction and in the direction of the courses determined for dry and wet relaxed rib knit fabrics after a domestic laundering and drying cycle.

All dry relaxed rib knitted fabrics showed shrinkage in the wale direction (Figure 3a), with a higher average shrinkage in the wale direction observed for modal (−18.8%) compared to modal-micro yarns (−16.5%) produced from ring-, open-end rotor- and air-jet-spun yarns. Shrinkage also occurred after the wet relaxation treatment (Figure 3b), but to a much lesser extent, which was acceptable and almost the same on average for modal (−8.1%) and modal-micro (−8.5%) rib knit samples produced from differently spun yarns.

This could be due to the fact that the incompletely balanced state of dry relaxed knitted fabric changes during the laundering and drying process when the structure of the knitted fabric changes. This is responsible for the shrinkage of knitted fabrics during laundering, whereby the tensions exerted on the fabrics during manufacture reappear during post-treatment processes if the fabric cannot fully relax. However, knitted fabrics also tend to exhibit residual extension.

In this respect, the dimensional changes observed in the course direction in dry relaxed rib knitwear are not uniform (Figure 3c), with knitwear made from ring-spun yarns expanding and knitwear made from open-end rotor-spun yarns shrinking. For dry relaxed knitwear made from air-jet-spun modal yarns, there is no change, but there is less shrinkage (for modal-micro knitwear, −3.3%). The dimensional changes in the knitted fabrics are caused by the relaxation of the yarns during laundering [49], and the resulting changes in the wale and course density of the knitted fabrics are described in Section 3.1. At the same time, the different dimensional changes in dry relaxed knitted fabrics after laundering are primarily due to different structural changes in the width direction of the knitwear (i.e., a change in wale count), which is directly related to the areal weight decrease in knitted fabrics made from ring-spun yarns, the increase in knitted fabrics made from open-end rotor-spun yarns and the smallest changes in knitted fabrics made from air-jet-spun yarns (Table 2).

In wet relaxed knitted fabrics with a balanced structure, the changes in the course direction are uniform and are shown by minimal shrinkage in most modal and modal-micro knitted fabrics (Figure 3d).

During use, the fiber material rubs off the surface of the knitted fabric, usually in direct contact with the user’s skin or other layers of clothing, causing fibers and yarns to fall out by splitting and pilling on the surface of the knitted fabric [50]. Therefore, if a one-by-one rib fabric (with equal surfaces on both sides) has a higher abrasion resistance and a lower tendency to pill on the surface, this has a positive effect on the durability of lingerie during use, but also on its surface appearance and esthetic properties.

The abrasion resistance of knitted fabrics is a very complex phenomenon and is influenced by many factors. Some of these affect the surface of the fabric, while others influence the internal structure of the fabric. The type of fibers and their fineness play an important role in surface abrasion, while the linear density of the yarn and the properties of the fabric structure are significantly related to structural abrasion [51].

The removal of fibers from the knitted structure is one of the reasons for abrasion. Therefore, the factors that influence the cohesion of the yarns also influence the abrasion resistance of the fabrics. The spinning process, the structure, the twist and the hairiness of the yarns are properties that also influence the abrasion resistance of knitted fabrics.

**Figure 4:**
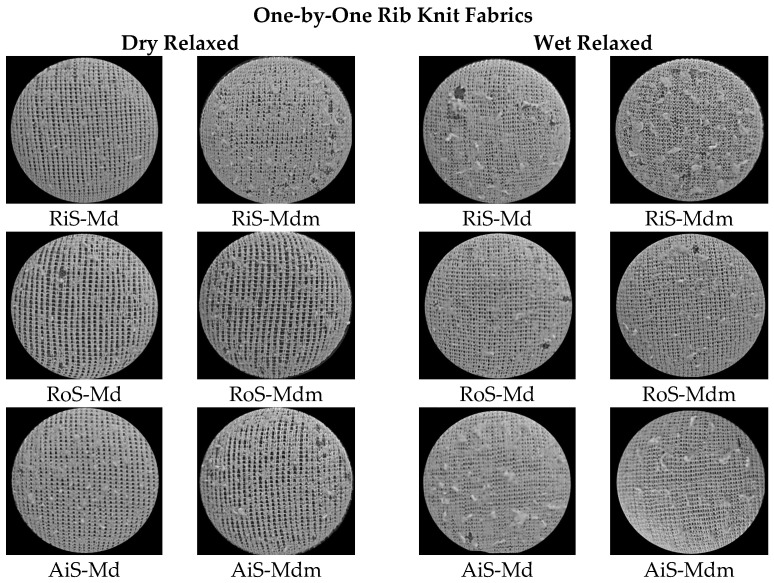
The appearance of the surface of modal (Md) and modal-micro (Mdm) dry relaxed (D) and wet relaxed (W) ribbed knitted fabrics fabricated from RiS (ring-spun), RoS (open-end rotor-spun) and AiS (air-jet-spun) yarns after the abrasion test.

**Figure 5:**
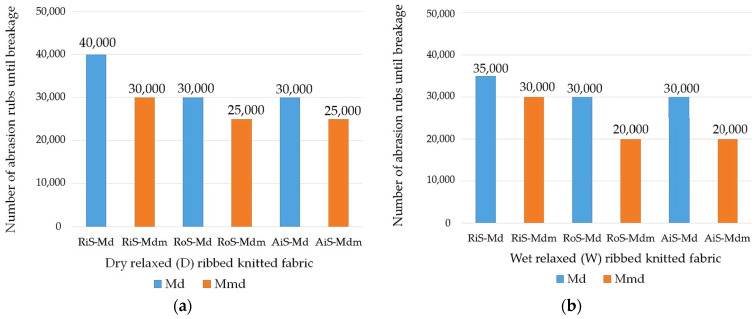
Number of abrasion rubs where breakage occurred in modal (Md) and modal-micro (Mdm) ribbed knitted fabrics fabricated from RiS (ring-spun), RoS (open-end rotor-spun) and AiS (air-jet-spun) yarns: (**a**) dry relaxed (D); (**b**) wet relaxed (W).

The appearance of the surface of the dry and wet relaxed rib fabrics at the end of the abrasion test and the corresponding number of abrasion rubs where breakage occurred are shown in Figure 4 and Figure 5, respectively.

In the comparative analysis of the abrasion properties of the knitted fabrics shown in Figure 5a,b, the highest values were determined for knitted fabrics made from ring-spun yarns, both for dry and wet relaxed samples. This was to be expected, considering that RiS yarns have the highest tenacity, the lowest overall unevenness (Table 1) and a tightly twisted surface structure that prevents the fibers from wearing out easily despite their higher hairiness. In addition, the number of abrasion rubs at break is lower in rib knitted fabrics made from open-end rotor- and air-jet-spun yarns with higher overall unevenness, lower tenacity and surface compactness. When evaluating the dry relaxed knitted fabrics made from these yarns from the same modal fibers or modal microfibers, the same abrasion values at which breakage occurred were determined for both (30,000 and 25,000, respectively).

It should be noted that all knitted samples of modal microfibers showed lower wear resistance than modal knitted fabrics of the same yarn type, both in the dry and wet relaxed samples, indicating easier surface wear of the microfibers in the ribbed knit structure, where the number of fibers in the yarn cross-section increases.

After a wet treatment (Figure 5b), however, the abrasion resistance of knitted fabrics made from modal ring-spun yarns decreases by 5000 abrasion rubs (from 40,000 to 35,000), whereby the knit structure expands in the course direction and the areal weight and thickness decrease. During the wet relaxation treatment, knitwear made from open-end rotor-spun yarn shrinks, the modal fibers adhere to the surface of the knitted fabric, so that the knitted fabric reaches a firmer state, and the movement of the fibers within the yarn is restricted. The result is a more stable, heavier and bulkier knitted structure (Table 2 and Table 3). Therefore, the abrasion resistance remains the same for knitwear made from modal RoS and AiS yarns (30,000 abrasion rubs). Another parameter that influences the abrasion resistance is the stitch density of the knitted fabric. The more stitches per unit area of the fabric, the lower the force on each thread.

The abrasion resistance of rib knitted fabrics made of ring-spun modal microfibers remains the same for wet relaxed knitted fabrics (30,000 abrasion rubs), but decreases for RoS and AiS knitted fabrics, with breakage occurring at 20,000 abrasion rubs, where the surface abrasion is higher despite the higher knit tightness, fullness and voluminosity.

The visual analysis of the abraded wet relaxed knitted fabric (Figure 4) shows more intensive and larger wear-related unevenness on the fabric surfaces compared to the dry relaxed knitted fabric. It follows that, after wet relaxation, only rib knitted fabrics made from ring-spun modal microfibers exhibit comparable abrasion properties to all knitted fabrics made from modal fibers, with the largest and densest interlaced clumps of drawn fibers being observed on their surface (Figure 4).

Detailed numerical pilling ratings after visual assessment of the dry and wet relaxed knitted fabric surfaces after a certain number of pilling rubs [44] are listed in Table 5. During the wear simulation, the occurrence of pilling is more pronounced in all rib knit samples tested with an increasing number of pilling rubs, so that the pilling rates are lower.

At the end of the test, after 7000 pilling rubs, the lowest pilling ratings were found for dry and wet relaxed rib knitted fabrics fabricated from ring-spun yarns, which then increased as follows: RiS knitted fabrics < RoS knitted fabrics < AiS knitted fabrics. The hairiness of the yarns used for rib knitting decreased as follows: RiS yarns > RoS yarns > AiS yarns. With hairiness values being lower for yarns made from modal microfibers (Table 1), it is obvious that knitted fabrics with lower surface hairiness have a lower tendency towards pilling.

Furthermore, in the production of open-end rotor-spun yarns, the core fibers that form the inner part of the yarn are twisted, while some of the fibers are wrapped around the already spun yarn, creating recognizable features (Figure 1). In air-jet yarns, the core fibers are also surrounded by wrapping fibers, while the core fibers are arranged parallel to the yarn axis without twisting and are periodically enclosed by the wrapping fibers (Figure 1) [13]. Despite the higher overall unevenness of the yarn, the entanglement of the fibers in the balls protruding from the rib knit of unconventionally spun open-end rotor and air-jet yarns is of lower intensity (Table 5).

The final pilling values are minimally reduced in the wet relaxed knitted fabrics (Table 5) with a modified structure of higher volume fulfillment and lower thickness (Table 2 and Table 3), but are well balanced in the rib knit fabrics made from the same yarn type. Rib knitted fabrics fabricated of air-jet-spun modal-micro yarn are the least susceptible to surface pilling and therefore have better esthetic properties (with a moderate pilling rate of 3.5 for the dry relaxed sample and 3 for the wet relaxed sample), confirming their usage durability.

#### 3.2.2. Wearing Comfort Properties of Knitted Fabrics

**Figure 6:**
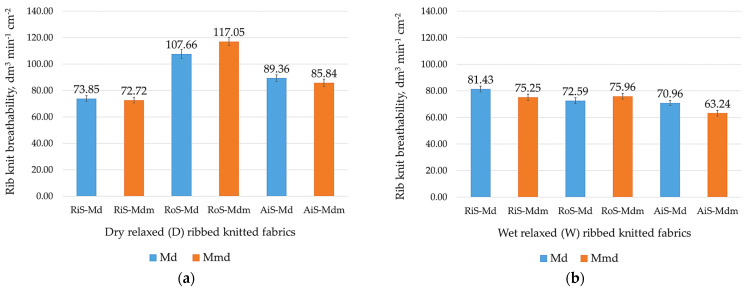
Breathability of ribbed knitted fabrics: (**a**) modal (Md) and modal-micro (Mdm) dry relaxed (D); (**b**) modal (Md) and modal-micro (Mdm) wet relaxed (W) ribbed knitted fabrics fabricated from RiS (ring-spun), RoS (open-end rotor-spun) and AiS (air-jet-spun) yarns.

**Figure 7:**
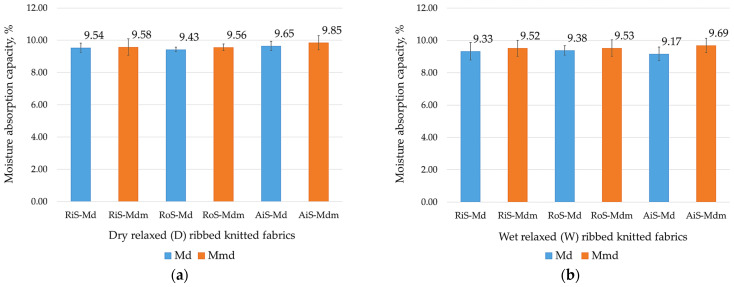
Moisture absorption capacity of ribbed knitted fabrics: (**a**) modal (Md) and modal-micro (Mdm) dry relaxed (D); (**b**) modal (Md) and modal-micro (Mdm) wet relaxed (W) ribbed knitted fabrics fabricated from RiS (ring-spun), RoS (open-end rotor-spun) and AiS (air-jet-spun) yarns.

Good breathability and the moisture absorption capacity of knitwear have a great influence on wearing comfort between lingerie and the skin in generally warmer microclimates with higher humidity [4,52]. Therefore, the values obtained for the dry and wet relaxed one-by-one rib knit fabrics, which were tested individually, are shown in Figure 6 and Figure 7.

Although it is generally assumed that the breathability of fabrics depends on their porosity [52], an important dependence of dense knitted fabrics on their structure, the type of yarn used, and the fineness of the fibers was found. As shown in Figure 6, the breathability of dry and wet relaxed ribbed knitted fabrics depends directly on the areal weight, thickness, structural density and voluminosity of the knitted fabric (Table 2 and Table 3). In this case, lighter and thinner knitted fabrics with a lower stitch density and lower volume have a higher breathability. It can be seen (Figure 6a) that the breathability of dry relaxed knitwear samples decreases as follows: RoS knitted fabric > AiS knitted fabric > RiS knitted fabric.

Due to the full relaxation of the knitted fabric after the wet treatment, the breathability of the wet relaxed knitted fabric is equalized; this it is mainly due to the volume fulfillment of the knitted fabric in addition to the changes in the physical properties of the densely structured knitted fabric (Table 3). This is particularly noticeable in modal-micro knitted fabrics, where the fineness of the microfibers causes the fibers to cluster very densely [1], and many finer fibers are needed to form the yarn fabricated from modal microfibers, which affects the greater voluminosity of the knitted fabrics. With the exception of the RoS-Mdm knitted fabric (Figure 6a,b), the air permeability values for all knitted fabrics made from modal microfibers are slightly lower compared to modal knitted fabrics.

Despite the fact that modal fibers have a relatively high moisture regain value (between 11.5% and 12.5% [5]) and all fibers used are made of the same polymer, the very densely packed microfibers form narrow capillaries, so that fabrics made of microfibers can absorb perspiration moisture more easily than those made of regular–fine fibers.

This can be seen from the results of the moisture absorption capacity of the dry and wet relaxed samples, which are shown in Figure 7. Higher moisture absorption was found for all knitted samples fabricated from microfibers (which is also consistent with data from the literature for single-jersey knitwear [1]). It should be noted that the moisture absorption capacity values of knitted fabrics fabricated from regular–fine modal fibers and microfibers are quite similar and that they decrease minimally after wet treatment as follows: for knitted fabrics fabricated from modal fibers, from −0.31% for RoS knitted fabric to −1.62% for AiS knitted fabric; and for knitted fabrics fabricated from modal microfibers, from −0.53% for RoS knitted fabric to −4.97% for AiS knitted fabric.

Air-jet yarns and knitted fabrics are said to have several unique properties that offer advantages over conventional yarn spinning technologies. These include lower hairiness and better pilling resistance [13], which was confirmed in this work, as well as higher moisture absorption, diffusion properties and faster drying. Despite very small mutual differences, it should be noted that there are inherent differences in moisture absorption between yarns produced using different spinning processes (Figure 7). Higher moisture absorption values were found for rib knitted fabrics fabricated from air-jet-spun yarns compared to RiS and RoS knitted fabrics (in the dry relaxed state for modal and modal-micro knitted fabrics and in the wet relaxed state for modal-micro knitted fabrics). Better moisture absorption leads to a stronger cold feeling in lingerie items [21], which is an advantage in warmer weather.

## 4. Conclusions

The progress in the application of innovative rib knitwear made of differently spun yarns of regular–fine modal fibers and microfibers at two different relaxation levels was comparatively analyzed. To address the problem of the insufficient use of modal fibers and microfibers in the production of lingerie, the applicability of which is confirmed by commercial use, the evaluated physical and wearing properties responsible for the usability, esthetics, and comfort of lingerie were discussed, and the following conclusions were drawn.

The relaxation phase of rib knit fabrics influences the results achieved. After the wet relaxation treatment, their structure reaches a balanced state and the physical properties of rib knitted fabrics change. Rib knitted fabrics made of modal microfibers become lighter and thinner; they have a higher voluminosity and consequently a slightly lower overall porosity than comparable fabrics made of modal regular–fine fibers. Knitted fabrics made from air-jet-spun yarns have the most stable structure.

In the comparative analysis, minimal differences in breaking strength and elongation at break were found when comparing the same knitted fabrics of modal fibers and microfibers in both dry relaxed and wet relaxed states. They depend primarily on the tenacity of the yarns used and the degree of balance of the knitted structure. Higher values of breaking strength and lower elongation were found in the lengthwise direction of the lingerie (highest for RiS knitted fabrics); in the widthwise direction it was the opposite, with elongation being much higher for rib knitted fabrics (highest for RoS knitted fabrics), making the lingerie easier to wear. The wet relaxation treatment reduces the tensile properties of all rib knitted fabrics.

Dimensional stability during the domestic care of wet relaxed rib knitwear of a balanced structure is considered acceptable. After a wash and dry cycle, modal and modal-micro knitwear showed shrinkage of up to −8.5% in the lengthwise direction, while shrinkage in the widthwise direction was lower for almost all samples (up to −3.3%), which has a positive effect on the durability of lingerie during use.

All knitted samples of modal microfibers showed lower abrasion resistance than modal knitted fabrics of the same yarn type, both in the dry and wet relaxed samples, indicating easier abrasion of the microfibers from the rib knit structure, with the highest resistance found in RiS knitted fabrics. After wet relaxation, only the rib knitted fabrics made from ring-spun modal microfibers exhibited comparable abrasion properties to all knitted fabrics made from modal fibers, albeit with more intense wear-induced unevenness on the surface. Consequently, the lowest pilling values were found for dry and wet relaxed rib knitted fabrics made from RiS yarns, with rib knitted fabrics made from air-jet-spun modal-micro yarn being the least susceptible to surface pilling, which strongly influences the appearance and esthetic properties of the knitted surface.

All rib knitwear samples made from modal microfibers were found to have a higher moisture absorption capacity, which means that they absorb perspiration moisture more easily than those made from regular–fine fibers and are more comfortable to wear. Higher moisture absorption values were found for rib knit fabrics made from air-jet-spun yarns. However, the breathability values of all rib knit fabrics made of modal microfibers, with the exception of the RoS-Mdm knit fabrics, were slightly lower compared to the modal rib knit fabrics, which was mainly due to the higher volume filling.

On the basis of the comparative analysis, it is concluded that the fiber, the yarn and the type of relaxation influence the properties of one-by-one rib lingerie fabrics. At the same time, rib lingerie fabrics made from modal microfibers are lighter and thinner, have a higher voluminosity, and have a higher moisture absorption capacity, and therefore exhibit slightly lower porosity, breathability and abrasion resistance than fabrics made from modal regular–fine fibers. They also have comparable dimensional stability, tensile strength and pilling properties, but mainly after wet relaxation treatment.

This means that the selection of knitted fabric must be adapted to the type of lingerie: knitted fabrics made of air-jet-spun yarns, which have better stability, contact comfort and esthetic properties, can be used for the production of finer women’s lingerie; knitted fabrics made of ring-spun yarns are recommended for lingerie with better mechanical properties and higher durability; and rib knitted fabrics made of modal-micro open-end rotor-spun yarn can be used for breathable lingerie with higher extensibility in width.

In our next study, the wear-related unevenness on the surfaces of the knitted fabrics will be examined in detail, as will the moisture-wicking properties of the knitted fabrics.

## Figures and Tables

**Table 1:** Physical–mechanical properties of modal (Md) and modal-micro (Mdm) differently spun yarns (RiS—ring-spun; RoS—open-end rotor-spun; AiS—air-jet-spun) used for knitting [16].

Spun Yarn	Yarn Linear Density, tex	Yarn Twist, m^−1^	Yarn Tenacity, cN/tex	Yarn Hairiness	Yarn Overall Unevenness, %
RiS-Md yarn	20.1 ± 0.16	751 ± 12.00	23.81 ± 1.64	6.09 ± 0.17	10.21 ± 0.10
RiS-Mdm yarn	20.1 ± 0.20	810 ± 14.60	24.09 ± 1.55	5.28 ± 0.21	9.67 ± 0.19
RoS-Md yarn	20.2 ± 0.17	*	15.39 ± 1.43	4.34 ± 0.08	13.95 ± 0.12
RoS-Mdm yarn	19.8 ± 0.17	*	15.86 ± 1.36	4.08 ± 0.07	12.69 ± 0.08
AiS-Md yarn	20.1 ± 0.06	**	20.77 ± 1.74	3.71 ± 0.17	12.33 ± 0.30
AiS-Mdm yarn	20.1 ± 0.09	**	20.55 ± 1.51	3.56 ± 0.24	12.12 ± 0.57

* Rotor speed, 753; ** Air pressure, 0.6 MPa.

**Table 2:** Areal weight, thickness, and number of wales and courses per cm of modal (Md) and modal-micro (Mdm) dry relaxed (D) and wet relaxed (W) ribbed knitted fabrics fabricated from RiS (ring-spun), RoS (open-end rotor-spun) and AiS (air-jet-spun) yarns.

One-by-One Rib Knit Fabrics	Areal Weight, g m^−2^		Thickness, mm		Number of Wales, cm^−1^		Number of Courses, cm^−1^	
	D	W	D	W	D	W	D	W
RiS-Md	162.5 ± 0.0	144.7 ± 0.0	0.84 ± 0.02	0.72 ± 0.01	20.0 ± 0.0	19.5 ± 0.5	12.0 ± 0.0	13.0 ± 0.0
RiS-Mdm	166.2 ± 0.0	140.9 ± 0.0	0.83 ± 0.01	0.68 ± 0.01	20.5 ± 0.5	19.0 ± 0.0	13.0 ± 0.0	13.0 ± 0.0
RoS-Md	143.8 ± 0.0	159.8 ± 0.0	0.79 ± 0.01	0.70 ± 0.01	18.5 ± 0.5	20.5 ± 0.5	13.0 ± 0.0	13.0 ± 0.0
RoS-Mdm	136.3 ± 0.0	156.9 ± 0.0	0.77 ± 0.01	0.68 ± 0.01	18.0 ± 0.0	19.0 ± 0.0	13.0 ± 0.0	13.0 ± 0.0
AiS-Md	150.3 ± 0.0	150.5 ± 0.0	0.85 ± 0.01	0.71 ± 0.01	19.0 ± 0.0	18.5 ± 0.5	12.5 ± 0.5	12.5 ± 0.5
AiS-Mdm	155.6 ± 0.0	148.6 ± 0.0	0.85 ± 0.01	0.65 ± 0.01	20.0 ± 0.0	20.0 ± 0.0	13.0 ± 0.0	12.0 ± 0.0

**Table 4:** Breaking strength and elongation at break of modal (Md) and modal-micro (Mdm) dry relaxed (D) and wet relaxed (W) ribbed knitted fabrics fabricated from RiS (ring-spun), RoS (open-end rotor-spun) and AiS (air-jet-spun) yarns.

One-by-One Rib Knit Fabrics	Breaking Strength, N				Elongation at Break, %			
	Wale Direction		Course Direction		Wale Direction		Course Direction	
	D	W	D	W	D	W	D	W
RiS-Md	313.7 ± 38.5	209.7 ± 23.5	93.6 ± 3.8	84.6 ± 1.8	44.3 ± 2.8	40.8 ± 1.2	150.0 ± 4.2	119.6 ± 1.8
RiS-Mdm	357.4 ± 28.3	220.3 ± 24.5	92.8 ± 3.2	83.3 ± 2.3	49.1 ± 3.4	36.2 ± 2.0	149.9 ± 6.5	109.4 ± 4.1
RoS-Md	261.0 ± 9.6	222.7 ± 15.9	79.0 ± 1.5	65.0 ± 0.8	41.2 ± 1.0	40.8± 2.4	182.1 ± 7.6	135.4 ± 3.2
RoS-Mdm	257.1 ± 14.5	228.9 ± 28.8	78.4 ± 1.8	66.5 ± 2.1	39.4 ± 2.0	43.7 ± 1.7	190.9 ± 3.7	139.3 ± 4.4
AiS-Md	292.3 ± 17.5	214.7 ± 26.7	82.6 ± 2.3	62.1 ± 3.9	42.0 ± 1.3	40.4 ± 0.5	175.4 ± 1.7	124.0 ± 4.0
AiS-Mdm	262.2 ± 23.7	208.8 ± 7.9	86.6 ± 2.0	59.4 ± 6.0	44.7 ± 1.7	40.1 ± 2.1	164.2 ± 4.3	120.9 ± 5.9

**Table 3:** Number of stitches per cm^2^, voluminosity and total porosity of modal (Md) and modal-micro (Mdm) dry relaxed (D) and wet relaxed (W) ribbed knitted fabrics fabricated from RiS (ring-spun), RoS (open-end rotor-spun) and AiS (air-jet-spun) yarns.

One-by-One Rib Knit Fabrics	Stitch Count, cm^−2^		Total Porosity, %		Voluminosity, g cm^−3^	
	D	W	D	W	D	W
RiS-Md	240.0	253.5	87.260	86.698	0.193	0.201
RiS-Mdm	266.5	247.0	86.791	86.302	0.200	0.207
RoS-Md	240.5	266.5	87.960	84.902	0.182	0.229
RoS-Mdm	234.0	247.0	88.336	84.525	0.177	0.234
AiS-Md	237.5	231.3	88.343	86.045	0.177	0.211
AiS-Mdm	260.0	240.0	87.881	84.625	0.184	0.233

**Table 5:** Visual evaluation of pilling on the surface of modal (Md) and modal-micro (Mdm) dry relaxed (D) and wet relaxed (W) ribbed knitted fabrics fabricated from RiS (ring-spun), RoS (open-end rotor-spun) and AiS (air-jet-spun) yarns after a certain number of pilling rubs.

One-by-One Rib Knit Fabrics	Dry Relaxed						Wet Processed					
	Number of Pilling Rubs											
	125	500	1000	2000	5000	7000	125	500	1000	2000	5000	7000
	Pilling Ratings											
RiS-Md	4.0	3.5	3.0	3.0	2.0	2.0	4.0	3.0	2.5	2.5	2.0	1.5
RiS-Mdm	4.5	3.5	3.0	3.0	2.5	1.5	4.5	3.5	3.0	2.5	2.0	1.5
RoS-Md	4.0	4.0	3.5	3.5	3.5	3.0	4.0	3.5	3.0	2.5	2.0	2.0
RoS-Mdm	4.5	4.0	4.0	4.0	3.0	2.5	4.5	4.0	3.5	3.0	2.5	2.0
AiS-Md	5.0	4.5	3.5	3.5	3.0	3.0	4.5	4.5	3.5	3.0	3.0	2.5
AiS-Mdm	4.5	4.5	4.5	4.0	3.5	3.5	4.5	4.0	4.0	3.5	3.0	3.0

## Data Availability

Data available in a publicly accessible repository.

## Abbreviations

The following abbreviations are used in this manuscript:

RiS	Ring-spun yarn
RoS	Open-end rotor-spun yarn
AiS	Air-jet-spun yarn
Md	Modal fibers
Mmd	Modal microfibers
RiS-Md yarn	Modal ring-spun yarn
RiS-Mdm yarn	Modal-micro ring-spun yarn
RoS-Md yarn	Modal open-end rotor-spun yarn
RoS-Mdm yarn	Modal-micro open-end rotor-spun yarn
AiS-Md yarn	Modal air-jet-spun yarn
AiS-Mdm yarn	Modal-micro air-jet-spun yarn
RiS-Md	Modal ring-spun knitted fabric
RiS-Mdm	Modal-micro ring-spun knitted fabric
RoS-Md	Modal open-end rotor-spun knitted fabric
RoS-Mdm	Modal-micro open-end rotor-spun knitted fabric
AiS-Md	Modal air-jet-spun knitted fabric
AiS-Mdm	Modal-micro air-jet-spun knitted fabric
D	Dry relaxed knitted fabric
W	Wet relaxed knitted fabric
